# Evaluating the Influence of Injury‐Induced Microstructural Variations on the Efficacy of a Diffusion MRI‐Derived Axon Diameter Surrogate

**DOI:** 10.1002/mrm.70415

**Published:** 2026-05-10

**Authors:** Hannah E. Alderson, Chaoqi Mu, Li Min Chen, Mark D. Does, Kevin D. Harkins

**Affiliations:** ^1^ Vanderbilt University Department of Biomedical Engineering Nashville Tennessee USA; ^2^ Vanderbilt University Institute of Imaging Science Nashville Tennessee USA; ^3^ Vanderbilt University Medical Center Department of Radiology and Radiological Sciences Nashville Tennessee USA; ^4^ Vanderbilt University Department of Electrical Engineering Nashville Tennessee USA

**Keywords:** axon diameter, diffusion, spinal cord injury

## Abstract

**Purpose:**

To investigate the utility of a recently demonstrated diffusion MRI derived axon diameter surrogate measure (∆*D*_⟂_) under pathologic conditions induced by a spinal cord injury model.

**Methods:**

Contusion injuries were performed on the thoracic–lumbar spinal cord of adult male rats. Eight weeks post‐injury, rats were sacrificed and their spinal cords imaged by MRI and then scanning electron microscopy (SEM). Relationships between the difference in radial diffusivity with diffusion time (∆*D*_⟂_) as well as longitudinal relaxometry based metrics and effective axon diameter from SEM were evaluated across regions of interest in slices 2–5 mm away from the injury epicenter.

**Results:**

∆*D*_⟂_ correlated strongly with histology derived effective axon diameter, while bound pool fraction and *T*_1_ of the free proton pool (*T*_1f_) had no significant correlations with effective axon diameter. A significant relationship was found between *T*_1f_ and ∆*D*_⟂_ across slices.

**Conclusion:**

In the context of contusion injured rat spinal cords, ∆*D*_⟂_ serves as a robust surrogate for axon diameter and provides complementary information to longitudinal relaxometry derived metrics.

## 1 Introduction

Axon diameter mapping using diffusion‐weighted MRI (dw‐MRI) has been an active area of research for two decades and is motivated by measuring changes in axon diameter that are associated with both healthy neurological development and pathology [1, 2, 3]. While numerous MRI methods exist for measuring axon diameter [4, 5, 6, 7, 8, 9], they are not without well‐known limitations that impede clinical translation. Specifically, many current approaches involve complex compartment modeling, extensive imaging protocols, and advanced hardware.

Along with those limitations, current models also show substantial bias in axon diameter estimates. For example, Sepehrband et al. found that even with strong preclinical gradients and ultra‐high field strength (16.4 T), diffusion MRI derived axon diameters overestimated values derived from histology (2–3 μm compared to ˜1 μm) [10]. As suggested by prior work [11, 12], this bias may arise from diffusion time dependence in the extracellular space that is not accounted for in the model. While many of the early methods assume diffusion time‐independence in the extracellular space [5, 6, 7], it is likely that both the intra and extracellular compartments contribute to observed diffusion time dependence [13, 14].

In a recent study, observed diffusion‐time dependent changes in radial diffusivity (∆*D*_⟂_) across the human corpus callosum at short and long effective diffusion times (∆_eff_) were attributed to changes in axon diameter [15]. Subsequent studies demonstrated a linear correlation between ∆*D*_⟂_ and axon diameter in simulation [16] and in ex vivo ferret spinal cords (SCs) [17]. This model‐free approach calculates ∆*D*_⟂_ by subtracting radial diffusivity measured at a long ∆_eff_ (*D*_⟂,PGSE_) with a pulsed gradient spin echo sequence (PGSE) from that measured at a short ∆_eff_ (*D*_⟂,OGSE_) using an oscillating gradient spin echo sequence (OGSE).

While not an absolute measure of axon diameter, ∆*D*_⟂_ offers advantages over previously published methods, including a modest acquisition protocol. In simulation, the relationship between ∆*D*_⟂_ and axon diameter was not impacted by changes in myelination, intercompartmental exchange, and extra‐axonal volume fraction. Additionally, though assumptions regarding the compartment contributions of diffusion time dependence have been demonstrated to bias current methods, ∆*D*_⟂_ was found to increase with axon size regardless of individual compartment contributions [16]. While the ex vivo experiments helped to validate the relationship between ∆*D*_⟂_ and axon diameter in healthy spinal cord tissue, further validation is needed, particularly in pathological conditions.

This study extends that earlier investigation of ∆*D*_⟂_ into a clinically relevant and well‐developed model for altering SC microstructure—a preclinical contusion spinal cord injury model [18, 19]. The pathophysiology of spinal cord contusion injury is complex, involving both primary injury of the tissue directly impacted, mixed with secondary injury mechanisms in surrounding regions [20]. Literature reports of pathology resulting from this model include neuronal degeneration [21], inflammatory responses [18], and demyelination [21, 22]. Previous work with this model has shown varying degrees of demyelination and remyelination several mm away from the injury at 8 weeks post contusion SC injury [23]. Using contusion spinal cord injury [18, 19] as a tool for altering microstructure in a clinically relevant way, this work demonstrates the efficacy of ∆*D*_⟂_ in reporting on axon diameter even when microstructural changes beyond axon diameter are present.

## 2 Methods

### 2.1 Spinal Cord Injury

All animal procedures were completed in compliance with NIH guidelines and approved by the Vanderbilt University Institutional Animal Care and Use Committee. SC injuries were performed as described in Mu et al. [18] on adult male Sprague Dawley rats (*N* = 4). Briefly, a laminectomy on the dorsal side of the spine was completed to expose the T12‐L1 SC segments. An impactor device created a moderate contusion injury at the midline of the L1 segment. Analgesics (5–15 mg/kg carprofen) and antibiotics (15–20 mg/kg Baytril) were administered in the post‐operative period.

### 2.2 Tissue Fixation

Eight weeks following injury, each rat was euthanized and perfusion fixed. Transcardial perfusions were completed using chilled phosphate‐buffered saline (PBS, 1×) with 47.6 mg of heparin and then with 2% PFA, 2.5% glutaraldehyde, 1× PBS, and 1 mM of gadolinium (ProHance). For 10 days, SCs were post‐fixed, and then rehydrated and stored long‐term in 1× PBS, 1 mM gadolinium (ProHance), and 0.01% sodium azide for ex vivo MRI data acquisition.

### 2.3 MRI Acquisitions

All MRI acquisitions were completed on a 15.2 T Bruker Biospec Avance III horizontal scanner (Billerica, MA) using the Bruker closed‐cycle helium cryoprobe (quadrature surface coil). Four slices were imaged on each SC at 2, 3, 4, and 5 mm above the center of the injury. At each slice two dw‐MRI acquisitions were completed—one at a short diffusion time (OGSE, ${∆}_{\mathrm{eff}}$ = 2.5 ms, one cosine period of 10 ms duration) and one at a long diffusion time (PGSE, ${∆}_{\mathrm{eff}}=$25 ms, ∆ = 26 ms, and *δ* = 3 ms). For both, the echo time (TE) = 40 ms, repetition time (TR) = 600 ms, *b* = 800 s/mm^2^, 1 *b* = 0 image, 15 diffusion encoded directions, and 8 averaged excitations (NEX). Additionally, a selective inversion recovery (SIR) [24] scan was completed with 12 inversion times, log‐spaced from 10 to 1500 ms, delay time = 600 ms, NEX = 20, and rapid acquisition relaxation enhancement (RARE) factor = 4. All acquisitions were completed with a slice thickness = 1 mm, field of view (FOV) = 3.3 × 3.3 mm^2^, encoding matrix = 44 × 44, and in‐plane resolution = 75 μm^2^. Total scan time was 10 h and 36 min per cord.

### 2.4 Scanning Electron Microscopy

The following scanning electron microscopy (SEM) processes were performed by the Vanderbilt Cell Imaging Shared Resource (CISR) core. Following the completion of MRI acquisitions, four slices measured from the center of the injury and matching the MRI slice locations from one SC were prepared for SEM using the TOU (tannic acid, osmium tetroxide, uranyl acetate) method described by Hart et al. [25]. After the TOU staining, samples were dehydrated in a graded ethanol series and infiltrated with Epon‐812 using propylene oxide as the transition solvent. Samples were polymerized for 48 h at 60°C. Sections were cut at a nominal thickness of 100 nm on a Leica UC7 ultramicrotome and collected on Si wafers. Entire cross‐sections of the SC were imaged via SEM and were performed on a Zeiss Crossbeam 550 operating at 5 keV, 500 nA using an annular backscatter detector. Atlas‐5 automation software was used to tile sections at 50 nm resolution, which was down‐sampled to 100 nm for analysis.

### 2.5 Data Analysis

Similar analysis methods to Alderson et al. [17] were completed in this work. In short, all MRI data were reconstructed and analyzed using MATLAB R2024b (The MathWorks, Natick, MA, USA). The REMMI toolbox [26] was used to perform diffusion tensor and quantitative magnetization transfer (qMT) analyses [24] on the diffusion tensor imaging (DTI) and SIR data, respectively. Resulting *D*_⟂,OGSE_, *D*_⟂,PGSE_, ∆*D*_⟂_, bound pool fraction (BPF), and *T*_1_ of the free proton pool (*T*_1f_) were used for further region of interest (ROI) analysis. Five ROIs—gracile fasciculus (FG), dorsal corticospinal tract (dCST), rubrospinal tract (RST), reticulospinal tract (ReST), and vestibulospinal tract (VST)—were selected based on a rat SC atlas [27] and previous work in thoracic rat SC [28]. Independent ROIs were drawn on the left and right sides of each SC, for a total of 10 ROIs per slice. Mean *D*_⟂,PGSE_, *D*_⟂,OGSE_, ∆*D*_⟂_, BPF, and *T*_1f_ were calculated for each of the 10 ROIs across all four slices for each of the four SCs (160 ROIs in total).

The same ROIs were selected from each SEM image and segmented into axon and myelin using AxonDeepSeg automatic segmentation [29]. Axon diameters were taken from the segmentation software, and area‐weighted mean inner axon diameters, ⟨*d*_eff_⟩, were calculated using MATLAB for each of the 10 ROIs across four slices. The equation used for ⟨*d*_eff_⟩ was chosen to match the axon diameter estimates in prior work [16, 17]. The right RST ROI of the 2‐mm slice was excluded due to the poor quality of histology in the region, resulting in 39 total ROIs for histology.

Linear regression was performed to evaluate relationships between ⟨*d*_eff_⟩ and *D*_⟂,PGSE_, *D*_⟂,OGSE_, ∆*D*_⟂_ (across slices for SC 1) as well as between ∆*D*_⟂_ and qMT derived metrics (across animals and slices). A multiple linear function of ⟨*d*_eff_⟩ and distance from the injury epicenter was also considered for radial diffusivity metrics. Parameters from linear regressions and Pearson's linear correlation coefficients were considered statistically significant for *p* values < 0.05.

## 3 Results

### 3.1 MRI Parameter Maps and SEM Images

**FIGURE 1:**
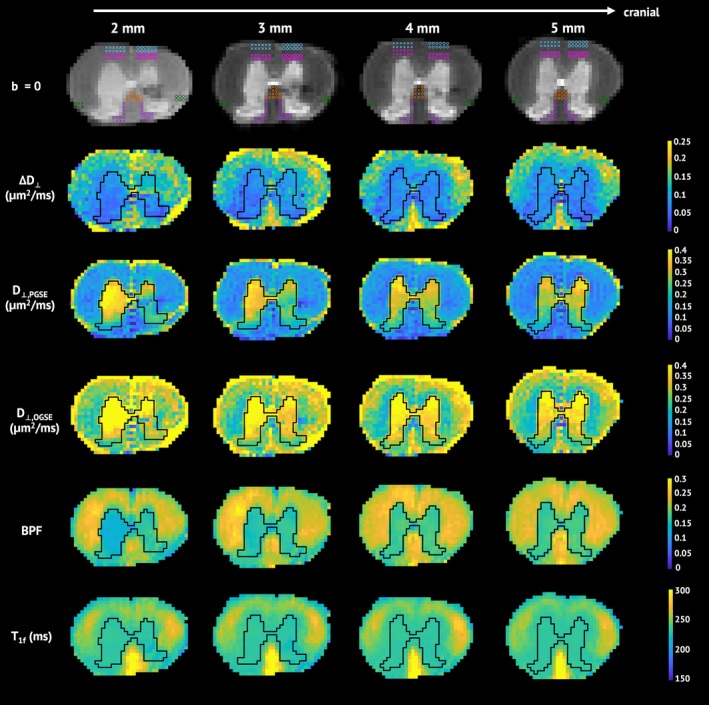
The top row shows the *b* = 0 images from the PGSE dataset for anatomic reference, including the ROIs selected for analysis. Purple is the gracile fasciculus (FG), orange is the dorsal corticospinal tract (dCST), green is the rubrospinal tract (RST), pink is the reticulospinal tract (ReST), and blue is the vestibulospinal tract (VST) Subsequent rows display each qMRI parameter map. Each column represents a slice location, moving further away from the injury epicenter from left to right.

Representative images and parameter maps from four spinal cord segments are shown for representative injured SC 1 in Figure 1. There is noticeable hyperintensity of the ∆*D*_⟂_, *D*_⟂,OGSE_, or *T*_1f_ maps in the medial FG region—the area of the spinal cord directly impacted by the injury. The right side of this specific spinal cord does appear to be more affected by the injury, resulting in noticeable left/right differences in contrast, including changes with slice location. ROIs selected for MRI analysis are highlighted by the x's (left) and o's (right) in the *b* = 0 images.

**FIGURE 2:**
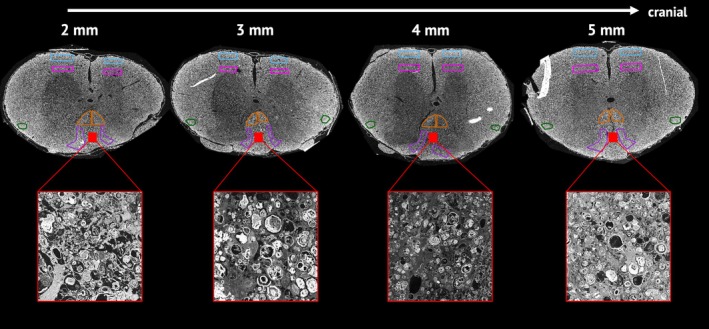
Scanning electron microscopy images of each slice for Spinal Cord 1. The ROIs are color coordinated to match Figure 1. Zoomed in portions of the medial FG are shown in the red outlined panels and display the severity of damage in that region due to injury. Each column represents a slice location, moving further away from the injury epicenter from left to right.

**FIGURE 3:**
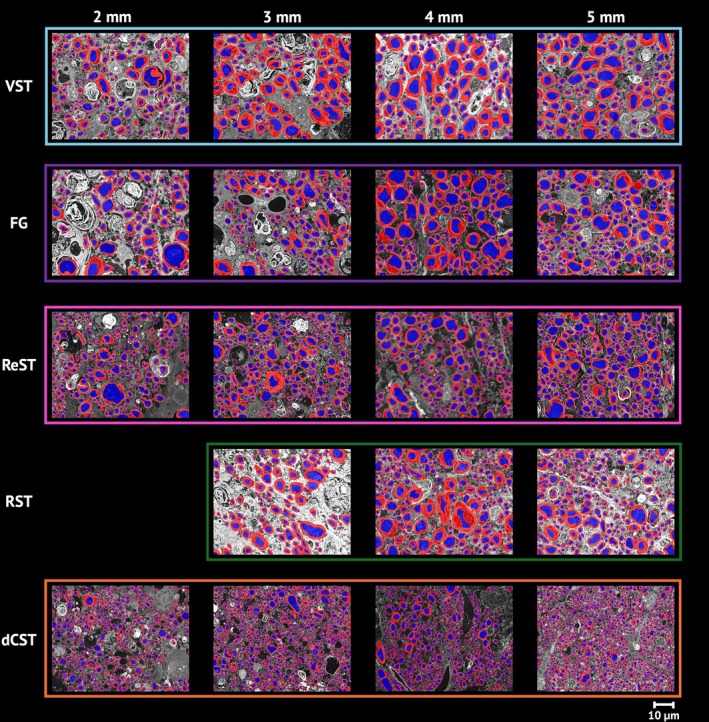
Zoomed in histology images for each right ROI at each slice location, segmented into axon (blue) and myelin (red). Each column represents a slice location, moving further away from the injury epicenter from left to right. Each ROI is outlined in the color that corresponds to Figures 1 and 2. As noted in the methods, RST for the 2 mm slice was of poor quality and not included.

Histology images shown in Figure 2 reveal a high level of damage from the injury, which highlights the extent of axonal damage in the medial FG. Segmentations were not reliable in this region and therefore only the lateral regions of the FG were included in further analyses. Zoomed‐in sections of the SEM images for the right ROIs are shown in Figure 3. Axon damage can be observed to varying degrees as the slices approach the injury center. The most apparent damage and changes in axon density are observed in the VST, lateral FG, and RST.

### 3.2 MRI Parameters and Histology Derived Axon Diameter

**FIGURE 4:**
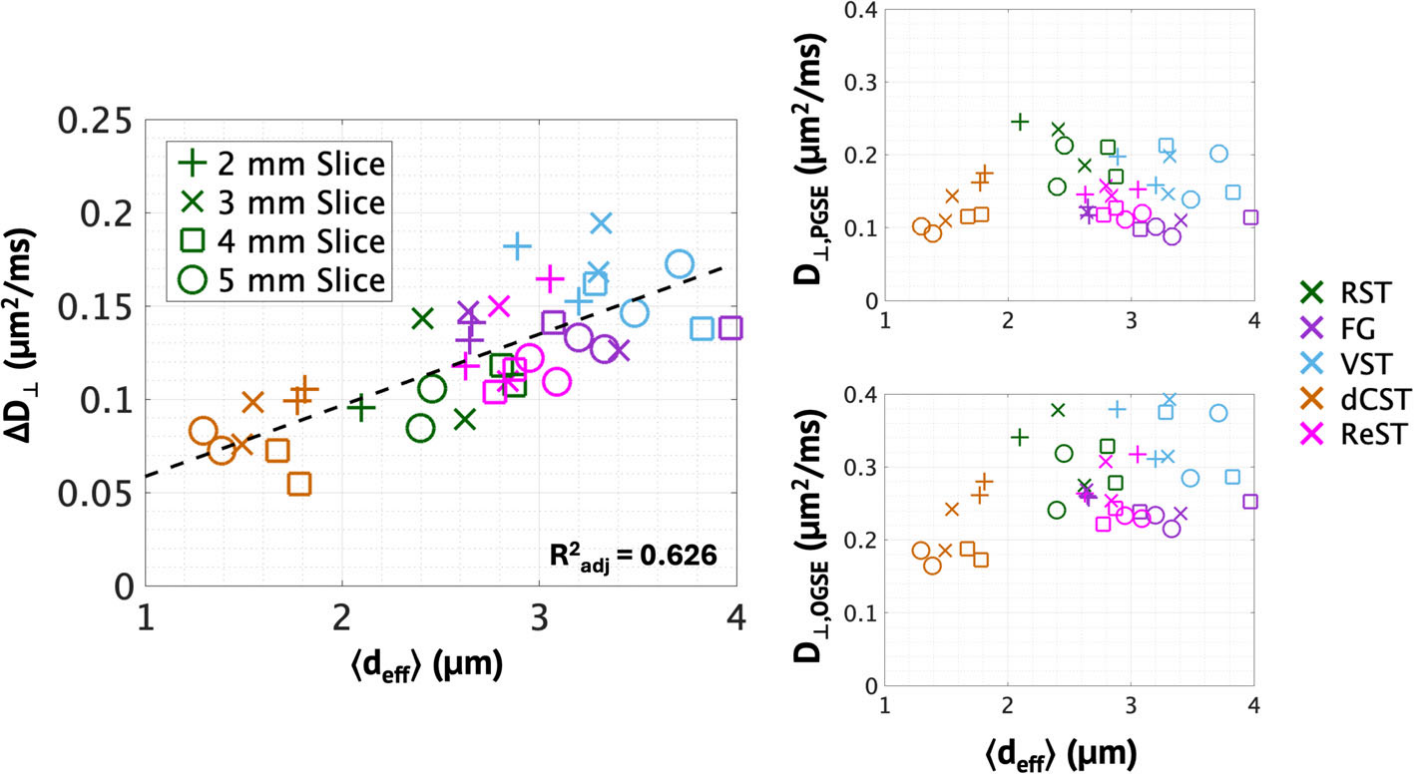
The relationship between ∆*D*_⟂_, *D*_⟂,PGSE_, *D*_⟂,OGSE_, and axon diameter derived from histology for each slice of spinal cord 1. The x's represent the data points on the right side of the spinal cord and the o's represent the left side. Each color represents the ROIs as indicated by the legend and corresponding to previous figures. The dashed line indicates the fitted model from the multiple linear regression.

Multiple linear modeling of ∆*D*_⟂_ demonstrated an improved fit compared to standard linear regression (likelihood ratio test *p* = 0.004, ∆AIC = 7.2). A significant positive association between ∆*D*_⟂_ and ⟨*d*_eff_⟩ across data from four slices of SC 1 was found (Figure 4, 𝛽_*d*eff_ = 0.053, *p* = 0.004). Distance from the injury epicenter and the interaction term were not significantly associated with ∆*D*_⟂_ (*p* = 0.894 and 0.331, respectively). Using the same multiple linear model, *D*_⟂,OGSE_ was not found to have a significant association with ⟨*d*_eff_⟩ with slice distance or the interaction term, though standard linear regression showed a significant linear correlation between *D*_⟂,OGSE_ and ⟨*d*_eff_⟩ (*R* = 0.451) No significant relationships were found between *D*_⟂,PGSE_ and ⟨*d*_eff_⟩ for any analyses. Summary statistics for the multiple linear model fitted parameters are reported in supplemental tables.

The VST was found to have the largest axons (3.09 ± 0.012 to 3.58 ± 0.016 μm), and correspondingly the largest ∆*D*_⟂_ values (0.149 ± 0.006 to 0.181 ± 0.005 μm^2^/ms), while dCST was found to have the smallest axons (1.34 ± 0.003 to 1.79 ± 0.004 μm), and the smallest ∆*D*_⟂_ values (0.066 ± 0.017 to 0.102 ± 0.016 μm^2^/ms). Values of ∆*D*_⟂_ and ⟨*d*_eff_⟩ are averaged across left and right ROIs and summarized in Table 1.

**TABLE 1:** MRI derived ∆*D*_⟂_ and effective axon diameter derived from SEM, averaged across regions of interest ± the standard error.

Slice location	ROI	∆*D*_⟂_ (μm^2^/ms)	⟨*d*_eff_⟩ (μm)
2 mm	VST	0.167 ± 0.008	3.09 ± 0.012
	FG	0.136 ± 0.006	2.65 ± 0.008
	ReST	0.141 ± 0.007	2.78 ± 0.011
	RST	0.095 ± 0.018	2.10 ± 0.015
	dCST	0.102 ± 0.016	1.79 ± 0.004
3 mm	VST	0.181 ± 0.005	3.31 ± 0.013
	FG	0.136 ± 0.007	3.06 ± 0.012
	ReST	0.130 ± 0.006	2.82 ± 0.012
	RST	0.116 ± 0.012	2.62 ± 0.017
	dCST	0.088 ± 0.013	1.52 ± 0.003
4 mm	VST	0.149 ± 0.006	3.57 ± 0.019
	FG	0.140 ± 0.006	3.56 ± 0.015
	ReST	0.110 ± 0.005	2.82 ± 0.012
	RST	0.112 ± 0.008	2.84 ± 0.016
	dCST	0.066 ± 0.017	1.72 ± 0.004
5 mm	VST	0.159 ± 0.008	3.58 ± 0.016
	FG	0.130 ± 0.005	3.27 ± 0.011
	ReST	0.116 ± 0.005	3.03 ± 0.011
	RST	0.095 ± 0.007	2.43 ± 0.011
	dCST	0.078 ± 0.025	1.34 ± 0.003

*Note*: These values are from spinal cord 1, with the different slice locations in reference to the center of the injury.

### 3.3 SIR‐Derived Myelin Measures and DTI‐Derived Axon Diameter Surrogate

**FIGURE 5:**
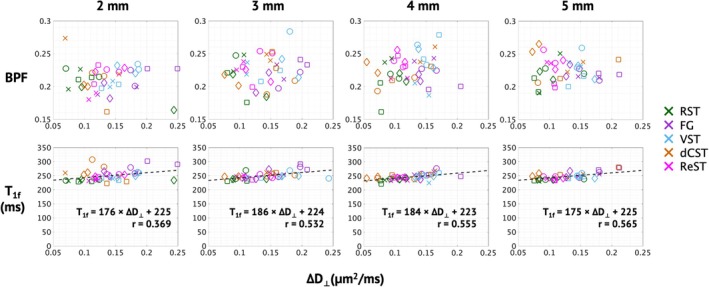
The relationship between BPF, *T*_1f_, and ∆*D*_⟂_ for each slice. Spinal Cord 1 (x), 2 (o), 3 (□), and 4 (♢) are all represented. Each color represents the ROIs as indicated by the legend and corresponding to previous figures. Trendlines (‐‐‐), slopes, intercepts, and correlation coefficients are displayed for significant correlations.

The correlations between ∆*D*_⟂_ and SIR‐derived metrics were evaluated across slices for all four SCs (Figure 5). Significant linear correlations were found between *T*_1f_ and ∆*D*_⟂_ across all slices (*r* = 0.369–0.565). Summary statistics for the linear regressions are reported in Table S3. There was no significant correlation between BPF and ∆*D*_⟂_ for any of the slices. MRI parameter values are averaged across SCs and ROIs, including the medial FG region where the primary damage was evident in histology, and are reported in Table 2. Overall, the damaged medial‐FG region showed higher ∆*D*_⟂_ (0.181 ± 0.057 to 0.201 ± 0.055 μm^2^/ms), *T*_1f_ (297 ± 14.3 to 312 ± 14.0 ms), and BPF (0.238 ± 0.008 to 0.252 ± 0.041) values compared to the other ROIs.

**TABLE 2:** MRI derived parameters (BPF, *T*_1f_, ∆*D*_⟂_) averaged across regions of interest and spinal cords (*N* = 4) ± the standard deviation for each slice location in reference to the center of injury.

Slice location	ROI	BPF	*T*_1f_ (ms)	∆*D*_⟂_ (μm^2^/ms)
2 mm	VST	0.215 ± 0.015	246 ± 10.4	0.160 ± 0.023
	FG	0.207 ± 0.018	267 ± 22.4	0.173 ± 0.042
	ReST	0.213 ± 0.019	245 ± 8.90	0.130 ± 0.021
	RST	0.207 ± 0.020	233 ± 2.76	0.113 ± 0.056
	dCST	0.203 ± 0.043	258 ± 27.3	0.122 ± 0.032
	FG mid	0.252 ± 0.041	297 ± 14.3	0.181 ± 0.057
3 mm	VST	0.255 ± 0.069	247 ± 9.76	0.164 ± 0.044
	FG	0.219 ± 0.018	264 ± 18.4	0.171 ± 0.028
	ReST	0.229 ± 0.019	245 ± 6.26	0.121 ± 0.022
	RST	0.232 ± 0.050	234 ± 12.4	0.103 ± 0.020
	dCST	0.223 ± 0.023	254 ± 6.24	0.139 ± 0.036
	FG mid	0.238 ± 0.008	300 ± 11.5	0.192 ± 0.019
4 mm	VST	0.237 ± 0.031	246 ± 11.0	0.149 ± 0.013
	FG	0.219 ± 0.017	255 ± 11.9	0.155 ± 0.024
	ReST	0.233 ± 0.015	242 ± 5.13	0.108 ± 0.010
	RST	0.213 ± 0.025	236 ± 6.82	0.095 ± 0.015
	dCST	0.225 ± 0.021	251 ± 10.1	0.111 ± 0.043
	FG mid	0.247 ± 0.019	302 ± 9.27	0.193 ± 0.034
5 mm	VST	0.227 ± 0.018	246 ± 5.98	0.148 ± 0.014
	FG	0.219 ± 0.008	259 ± 13.1	0.163 ± 0.029
	ReST	0.229 ± 0.019	239 ± 5.40	0.106 ± 0.010
	RST	0.216 ± 0.020	235 ± 3.41	0.100 ± 0.026
	dCST	0.240 ± 0.026	255 ± 12.4	0.109 ± 0.057
	FG mid	0.250 ± 0.011	312 ± 14.0	0.201 ± 0.055

### 3.4 Longitudinal Diffusivity

For completeness, longitudinal diffusivity (*D*_||_) maps are shown in Figure S1. Low *D*_||,PGSE_ and *D*_||,OGSE_ values are observed in the medial‐FG region. Similar to the radial diffusivity maps, there are noticeable left/right differences in contrast, with lower *D*_||,PGSE_ and *D*_||,OGSE_ on the right side. Unlike ∆*D*_⟂_, there is no obvious hyperintensity in the medial‐FG region of the ∆*D*_||_ maps across slices compared to the rest of the spinal cord.

## 4 Discussion

This study evaluated the efficacy of ∆*D*_⟂_ in reporting on axon diameter under pathologic conditions, namely the acute phase of traumatic spinal cord injury. Across 39 ROIs and four SEM slices, ∆*D*_⟂_ was strongly associated with histology derived mean axon diameter (𝛽_*d*eff_ = 0.053, *p* = 0.004). It is worth noting that given the heterogeneity expected in injury across animals, the sample size of *N* = 4 slices from one SC for histology validation is a limitation of current analyses. Additionally, qMT derived measures that are commonly used to report on myelin content were also considered. No significant correlations were found between BPF and ∆*D*_⟂_; however, a significant positive correlation was found between *T*_1f_ and ∆*D*_⟂_ across slices and spinal cords.

Previous research demonstrated strong correlations between both ∆*D*_⟂_ and *D*_⟂,OGSE_ and ⟨*d*_eff_⟩ (*r* = 0.923 and *r* = 0.912, respectively) [17]. Simulations suggested ∆*D*_⟂_ offers an advantage over *D*_⟂,OGSE_ by more robustly reporting on axon diameter when changes to the extra‐axonal volume fraction, myelination, and axon size distribution are present [16]. This is supported by the findings presented here, which showed no significant association between *D*_⟂,OGSE_ and ⟨*d*_eff_⟩ in the presence of injury‐induced microstructural variations. Furthermore, while changes in *D*_⟂,PGSE_ with axon diameter were suggested in simulation to be largely driven by changes in the extra‐axonal space, there was no correlation between *D*_⟂,PGSE_ and ⟨*d*_eff_⟩ in injured tissue shown here, or healthy tissue demonstrated previously [17].

While the strong association between ∆*D*_⟂_ and ⟨*d*_eff_⟩ initially demonstrated in healthy spinal cord is retained here in injured spinal cord, it is important to acknowledge that the impact of individual injury mechanisms was not evaluated. Edema, gliosis, inflammation, demyelination/remyelination, and axonal beading are all expected to occur as a result of spinal cord injury. The histological analysis did not assess these individual mechanisms, and therefore a statement regarding the specific or individual impact of each on the ∆*D*_⟂_/⟨*d*_eff_⟩ relationship cannot be made. However, it is expected that primary impacts of these injury mechanisms are canceled out in the subtraction between *D*_⟂,OGSE_ and *D*_⟂,PGSE_ [16]. Additionally, the slope found for the ∆*D*_⟂_/⟨*d*_eff_⟩ relationship in healthy spinal cord was 0.065 [17], which is comparable to that reported here (0.053).

In the healthy ferret spinal cord, ∆*D*_⟂_ was found to range from ˜0.15 to 0.35 μm^2^/ms and corresponded to a range in ⟨*d*_eff_⟩ of ˜2–5 μm [17]. Here, ∆*D*_⟂_ was found to range from ˜0.05 to 0.2 μm^2^/ms and corresponded to a range in ⟨*d*_eff_⟩ of ˜1–4 μm. The shift in ∆*D*_⟂_ to a smaller range in the rat spinal cord evaluated here is accompanied by a smaller range in axon diameter, as expected.

Previous work found a negative correlation between *R*_1f_ and axon diameter in healthy rat spinal cord [30]. Authors of that work proposed that these trends were due to either surface relaxation effects between intra/extra axonal water and myelin, or due to myelin mediating water exchange between intra and extra‐axonal compartments. Where in either case, a longer *T*_1f_ would be observed for bigger/more myelinated axons. If ∆*D*_⟂_ does reflect axon size, the results in Figure 5 are in good agreement with prior experiments; however, a limitation of current work is that histology data was only acquired for four slices from SC 1 [30].

In addition to the quantitative information presented in this work, the contrast in ∆*D*_⟂_, *D*_⟂,OGSE_, and *T*_1f_ parameter maps was especially sensitive to the region of impact from the contusion injury. All three parameters were noticeably elevated in the medial FG (highlighted in Figure 2) compared to the rest of the spinal cord (Figure 1). ∆*D*_⟂_ is only expected to report on axon diameter in ordered white matter and therefore is not informative as an axon diameter surrogate in highly degenerated tissue. Though segmentations of these regions were not considered reliable enough to include in analyses, the contrast provided by the radial diffusivity and relaxometry parameter maps indicated sensitivity to the extensive damage in that region. Furthermore, in the same region, the longitudinal diffusivity (*D*_||_) maps (Figure S1) from both PGSE and OGSE are noticeably lower compared to other regions of the spinal cord. Decreases in *D*_||_ are often attributed to neuronal degeneration [31, 32], while changes in *D*_⟂_ are associated with changes in myelination [31, 33, 34]. The respective time‐dependent difference measures (∆*D*_||_ and ∆*D*_⟂_) also appear to be impacted differently from one another by injury. Contrary to ∆*D*_⟂_, ∆*D*_||_ does not show hyperintensity in the injured medial FG region compared to the rest of the spinal cord.

The focus of this work was not on investigating the injury itself but rather using the injury model as a clinically relevant tool for investigating ∆*D*_⟂_; however, future work should entail applying ∆*D*_⟂_ to directly investigate injury or disease models where axon diameter changes are expected. ∆*D*_⟂_ may serve as a robust surrogate for axon diameter, providing complementary information to existing markers, such as BPF, and overall improved interpretations of qMRI changes in disease models. Importantly, ∆*D*_⟂_ may provide a non‐invasive measure of axon diameter where histology is not possible. For example, work completed by Totoiu and Keirstead demonstrated chronic and progressive demyelination over a period of 450 days post spinal cord injury [22]. These experiments were performed in rats and included histology at several time points over 450 days. ∆*D*_⟂_ may enable similar investigations, along with myelin qMRI, to study spinal cord injury pathology in vivo animal models and in human subjects without the need for histology.

This work was performed on a high‐field, 15.2 T, preclinical MRI system. Diffusion acquisitions were optimized to maximize ∆*D*_⟂_ and therefore sensitivity to axon size for this specific hardware. The maximum *b*‐value that could be obtained at the short diffusion time was 800 s/mm^2^; however, higher *b*‐values were not needed as the framework for ∆*D*_⟂_ assumes a low *b*‐value regime. Additionally, while protocols in this work are not directly translational to clinical scanners, prior work has demonstrated the feasibility of this measurement in a more clinically relevant setting [15]. Authors collected data at 4.1 ms and 40 ms, *b* = 300 s/mm^2^, and were able to detect differences in ∆*D*_⟂_ along the human corpus collosum and between sexes using a 4.7 T Varian scanner. Nonetheless, future work will include optimization for clinical acquisitions to demonstrate the translatability of ∆*D*_⟂_.

Additionally, it is worth noting that effective diffusion times are not well defined for all diffusion waveforms. The oscillating waveform used here does have a widely accepted diffusion time as described in Parsons et al. [35] Even still, diffusion data acquired at the same reported diffusion time with two different waveforms (e.g., oscillating vs. pulsed) may not have the same values of radial diffusivity.

Toward the application of ∆*D*_⟂_ to injury models, ∆*D*_⟂_ provides benefits over existing axon diameter estimation methods. Namely, it is model‐free, making no assumptions about the compartmental contributions to the observed signal. This is particularly useful as appropriate assumptions regarding diseased tissue are often not known and is where current methods have limited utility. Additionally, it is clinically feasible to acquire single‐shell, low *b*‐value diffusion scans at two different effective diffusion times, as described above. Lastly, ∆*D*_⟂_ is sensitive to both intra‐ and extra‐axonal diffusion, whereas current methods may experience bias due to assumptions about compartment contributions [1, 11, 36].

As mentioned in the introduction, previously established methods tend to overestimate axon diameter even with strong gradients and high field strength [10]. Unlike other measures, ∆*D*_⟂_ does not provide axon diameter values; rather, it is reflective of axon size in that an increase or decrease in ∆*D*_⟂_ can be inferred as a corresponding shift in mean axon size. Therefore, a relevant measure of the accuracy of ∆*D*_⟂_ is the root mean squared error, which is 0.02 μm^2^/ms for the data presented in Figure 4. Furthermore, while compartment biases are not expected to impact ∆*D*_⟂_, it is possible other aspects of microstructure are not accounted for in this framework and may lead to biases.

Current validation work for ∆*D*_⟂_ is limited to ex vivo healthy and contusion injured spinal cords. Translation of ∆*D*_⟂_ as an axon diameter surrogate to other tissues and pathologies should be thoroughly explored. Axons in the spinal cord are simply organized, mostly parallel to one another. In the brain there are many regions of crossing fibers, and this may impact the relationship between ∆*D*_⟂_ and axon diameter. Specifically, it would be expected that with increasing axonal dispersion, diffusion behavior would be closer to isotropic diffusion and exhibit less diffusion time dependence. Nonetheless, there are regions of the brain where fibers are minimally dispersed, and ∆*D*_⟂_ may still serve as an axon diameter surrogate.

Finally, future work should also include optimizing ∆*D*_⟂_ studies for in vivo experiments. In vivo measurements are inherently noisier than ex vivo, more prone to artifacts, and have increased constraints on scan time; however, as previously mentioned, ∆*D*_⟂_ measurements in healthy human subjects have already been demonstrated [15], suggesting that with appropriate protocol design—such as improved sequence optimization, robust artifact correction, and careful motion compensation—these challenges can be addressed.

## 5 Conclusions

In the context of contusion spinal cord injury induced changes in microstructure, ∆*D*_⟂_ proves to be a robust surrogate for histology measured axon diameter. Additionally, the lack of correlation between BPF and ∆*D*_⟂_ indicates that ∆*D*_⟂_ provides complementary information to the myelin measure in evaluating disease.

## Funding

This work was supported by the National Institutes of Health (RO1 EB031954).

## Conflicts of Interest

The authors declare no conflicts of interest.

## Supporting information


**Figure S1:** The top row show the *b*‐val = 0 images for anatomic reference, including the ROIs selected for analysis. Subsequent rows display each longitudinal diffusion parameter maps. Each column represents a slice location, moving further away from the injury epicenter from left to right.
**Table S1:** Summary statistics for each of the fitted parameters from the multiple linear regressions evaluating the relationships between ∆*D*_⟂_ and *d*_eff_, and *D*_⟂,OGSE_ and *d*_eff_ displayed in Figure 4.
**Table S2:** Summary statistics for each of the fitted parameters from the standard linear regressions evaluating the relationships between ∆*D*_⟂_ and *d*_eff_, and *D*_⟂,OGSE_ and *d*_eff_ displayed in Figure 4. As described in the text, multiple linear analysis provided lower AIC and therefore a better fit of ∆*D*_⟂_.
**Table S3:** Summary statistics for each of the fitted parameters from the linear regressions evaluating the relationships between *T*_1f_ and *d*_eff_ displayed in Figure 5.

## Data Availability

The data that support the findings of this study are available from the corresponding author upon reasonable request.
