# Supplementary material for: Evaluating the Influence of Injury‐Induced Microstructural Variations on the Efficacy of a Diffusion MRI‐Derived Axon Diameter Surrogate
Source: Magn Reson Med. 2026 May 10;96(3):1355–64. doi: 10.1002/mrm.70415 (PMC13327435; doi:10.1002/mrm.70415)
Supplement: Supplementary file 1 — Figure S1: The top row show the b‐val = 0 images for anatomic reference, including the ROIs selected for analysis. Subsequent rows display each longitudinal diffusion parameter maps. Each column represents a slice location, moving further away from the injury epicenter from left to right. Table S1: Summary statistics for each of the fitted parameters from the multiple linear regressions evaluating the relationships between ∆D ⟂ and d eff, and D ⟂,OGSE and d eff displayed in Figure 4. Table S2: Summary statistics for each of the fitted parameters from the standard linear regressions evaluating the relationships between ∆D ⟂ and d eff, and D ⟂,OGSE and d eff displayed in Figure 4. As described in the text, multiple linear analysis provided lower AIC and therefore a better fit of ∆D ⟂. Table S3: Summary statistics for each of the fitted parameters from the linear regressions evaluating the relationships between T 1f and d eff displayed in Figure 5. [file MRM-96-1355-s001.docx]

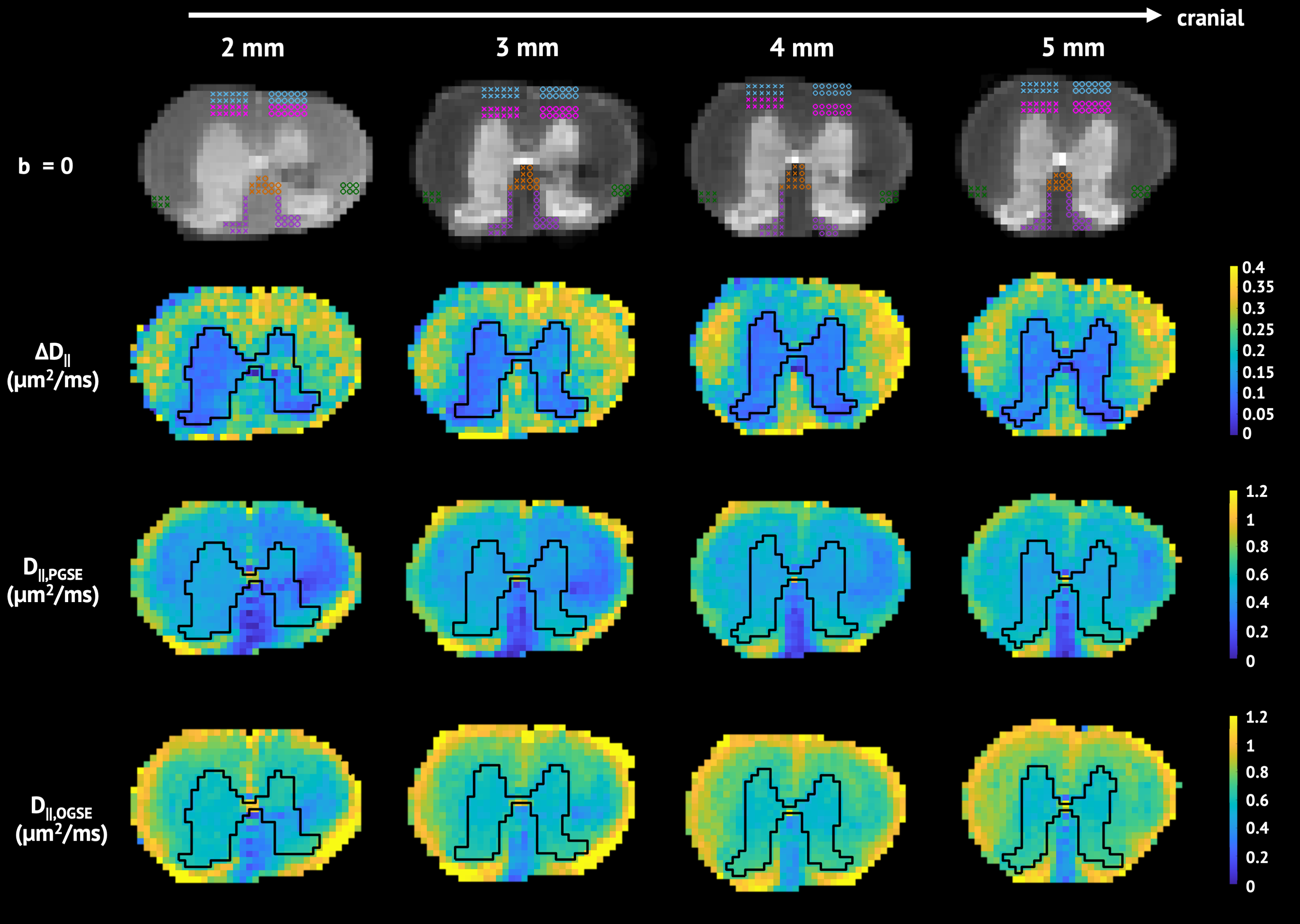


**Figure S1 – The top row show the b-val = 0 images for anatomic reference, including the ROIs selected for analysis. Subsequent rows display each longitudinal diffusion parameter maps. Each column represents a slice location, moving further away from the injury epicenter from left to right.**

**Table S1 – Summary statistics for each of the fitted parameters from the multiple linear regressions evaluating the relationships between ∆D_⟂_ and d_eff_, and D_⟂,OGSE_ and d_eff_ displayed in Figure 4.**

**Table S2 – Summary statistics for each of the fitted parameters from the standard linear regressions evaluating the relationships between ∆D_⟂_ and d_eff_, and D_⟂,OGSE_ and d_eff_ displayed in Figure 4. As described in the text, multiple linear analysis provided lower AIC and therefore a better fit of ∆D_⟂._**

**Table S3 – Summary statistics for each of the fitted parameters from the linear regressions evaluating the relationships between T_1f_ and d_eff_ displayed in Figure 5.**
